# Comparative study between partial and complete lateral anal sphincterotomy in treatment of chronic anal fissure and its effect on fecal incontinence

**DOI:** 10.1007/s10151-026-03343-3

**Published:** 2026-05-24

**Authors:** Ahmed Maher Abd Elmonim, Ahmed Eid Aziz, Mohamed H. Fahmy, Farid Mokhtar Farid Mohamed Abouelhoda, Mahmoud Azhary

**Affiliations:** Faculty of Medicine, Cairo University, Cairo, Egypt

**Keywords:** Chronic anal fissure, Complete lateral anal sphincterotomy, Fecal incontinence, Partial lateral anal sphincterotomy

## Abstract

**Background:**

Chronic anal fissure is a painful condition that can greatly affect quality of life. When conservative treatment fails, lateral internal anal sphincterotomy is performed to promote healing. The procedure may involve partial or complete sphincter division, which can influence the risk of fecal incontinence. This study aimed to compare the outcomes of tailoring the length of the lateral internal anal sphincter with either the whole length or the fissure apex and their effect on fecal continence in the treatment of chronic anal fissure.

**Methods:**

This comparative, prospective, observational study included 40 adults with chronic anal fissures that did not improve with medical treatment. Patients were divided into two equal groups on the basis of the type of sphincterotomy performed: full-length sphincterotomy or sphincterotomy limited to the fissure apex. Patient details and postoperative outcomes were closely monitored, with follow-up visits conducted over 1 month to assess recovery and complications.

**Results:**

The present study revealed significant differences between the studied groups in terms of postoperative pain and bleeding occurrence (*P* < 0.001). Postoperative pain was significantly higher among patients who underwent complete sphincterotomy (70%) compared with those who underwent partial sphincterotomy (20%). Similarly, bleeding was significantly more common in the complete sphincterotomy group (55%) than in the partial sphincterotomy group (25%).

**Conclusions:**

Partial lateral internal anal sphincterotomy appears to offer a balance between fissure healing and continence preservation, making it a preferable option for patients at higher risk of incontinence.

**Trial registration:**

Not applicable.

## Introduction

An anal fissure is a painful cut or crack in the distal anal canal. The acute form of the disease responds readily to nonsurgical methods, such as dietary bran supplements, warm sitz baths, and/or topically applied local anesthetic creams [1]. The characteristic skin tag and anal papilla are also regarded as indicators of poor response to conservative therapy [2].

Therefore, surgical treatment is almost uniformly recommended for such fissures in the chronic state, lateral internal anal sphincterotomy (LIS) being the time-honored treatment. LIS lowers the pressure exerted by the internal anal sphincter (IAS), restores normal perfusion of the anoderm, and leads to relief of pain and healing of the fissure [3].

Remaining as the gold-standard treatment of chronic anal fissure (CAF), use of LIS has been increasingly discouraged because of the significant rates of postoperative anal incontinence [4]. Although most episodes are minor and transient, impairment of continence in up to 45% of patients has been reported [5].

To alleviate postoperative disturbances of continence, tailoring of the height of the sphincterotomy to the length of the fissure has been suggested as an alternative to dogmatic division of the IAS to the dentate line, which is quoted as standard practice in most surgical texts [6].

Womack et al. [7] categorized incontinence into continence to solid/liquid stool and flatus (grade A), continence to solid and liquid stool but not to flatus (grade B), continence to solid stool but not to liquid or flatus (grade C), and finally incontinence to solid/liquid stool and flatus (grade D) [8]. This study aimed to compare the outcomes of tailoring the length of the lateral internal anal sphincter to either the entire length or to the fissure apex and their effect on fecal continence in the treatment of chronic anal fissure.

## Methods

This study was a comparative, prospective, observational analysis conducted on 40 patients aged from 18 to 50 years, diagnosed with chronic anal fissures (≥ 6 weeks duration) unresponsive to conservative treatment (e.g., topical nitrates or botulinum toxin injections), no history of previous anal surgery (except for fissure-related interventions), no history of major pelvic or colorectal surgeries, and ability to provide informed consent and participate in follow-up assessments. This study was conducted at the Multicenter, including the Faculty of Medicine, Cairo University, during the period from March 2024 to January 2025. The study was explained to the patients, and written informed consent was obtained from them before their enrollment in the study.

These patients were divided into two groups on the basis of the type of sphincterotomy performed: group A, which had lateral anal sphincterotomy (LAS) along the entire length of the internal anal sphincter (*n* = 20), and group B, which had LAS up to the fissure apex (*n* = 20).

Exclusion criteria were as follows: patients at the extremes of age (age < 18 years and > 50 years); patients with previous anal operations or prolapse; pregnancy or lactation; coexisting conditions such as inflammatory bowel disease, rectal cancer, or neurological disorders affecting anal sphincter function; active infection or other contraindications to surgery; and uncontrolled systemic diseases, such as diabetes mellitus or hypertension, which could affect wound healing.

The following demographic data were collected for all patients: age; gender; associated comorbidities; previous surgeries; time of presentation; preoperative investigations; postoperative follow-up for all cases; and vital signs: heart rate, blood pressure, respiratory rate (RR), temperature, and complete blood count (CBC) (performed on day 1 post-operation [PO]). Alarming signs to be monitored included: tachycardia, blood pressure drop, elevated temperature, or hemoglobin drop. Clinical follow-up was scheduled at 1 week, 2 weeks, and 1 month postoperatively. In addition, radiological investigations included abdominal computed tomography (CT) scan or endoscopy to confirm whether bleeding or leakage occurred, despite initial resuscitation measures. The rates of postoperative blood transfusion, hospital stay, and readmission were recorded.

### Surgical technique

- Partial LIS: In this procedure, a partial division of the internal anal sphincter (IAS) was performed, typically limited to the area of the fissure, without involving the full circumference of the sphincter.
- Complete LIS: Complete division of the internal anal sphincter was carried out, typically up to the dentate line, to ensure adequate reduction in anal sphincter tone. Both procedures were performed under local anesthesia, with the patient placed in the lithotomy position.

### Outcome measures

- Primary outcome: Postoperative fecal incontinence, categorized into incontinence to flatus, liquid stool, and solid stool. This was evaluated using a standardized fecal incontinence questionnaire and clinical assessment.
- Secondary outcomes: Fissure healing (evaluated through clinical examination at 6 weeks and 6 months postoperatively), postoperative pain (measured using a visual analog scale [VAS]), postoperative bleeding (measured as the percentage of patients experiencing bleeding after surgery), complications (e.g., infection, abscess, or anal stricture), and patient satisfaction with surgery, assessed through a follow-up questionnaire.
- Follow-up: All patients were followed up at 1, 4, and 6 weeks, and then at 6 months postoperatively for assessment of fissure healing, continence status, and any complications. Patients were advised on dietary modifications, stool softeners, and proper hygiene during the postoperative period.

### Statistical analysis

All data were compiled and analyzed using the Statistical Package for the Social Sciences (SPSS; version 21) (SPSS Inc., Chicago, IL, USA). Continuous variables are presented as means (± standard deviation [SD]), and categorical variables are presented using relative frequency distributions and percentages. Continuous variables were compared using Student’s *t*-test or the Mann–Whitney (*U*) test, and categorical data were analyzed using the chi-squared (*χ*^2^) test. Fisher’s exact test was used to gauge whether there was a statistically significant difference between the proportions of the categories in two group variables. They represent both standard ranges and optimal health ranges. *P* ≤ 0.05 and *P* ≤ 0.01 were used to determine whether the results were significant or very significant.

## Results

A total of 40 patients were included in this study; most of them were female individuals (*n* = 24), with 13 patients undergoing partial sphincterotomy and 11 patients undergoing complete sphincterotomy. The age of the studied patients ranged from 18 to 50 years, with a mean of 40.6 ± 7.80 years in patients undergoing partial sphincterotomy and 42.85 ± 5.78 years in patients undergoing complete sphincterotomy. There were no significant differences between the studied groups in terms of patient gender and age (*P* > 0.05). Additionally, there were no significant differences between the studied groups in terms of gender and medical history (*P* > 0.05). Additionally, pain was the most common among both groups (*n* = 8, 40%), and bleeding with defecation was more frequent among patients with partial sphincterotomy (*n* = 4, 20%) versus patients with complete sphincterotomy (*n* = 2, 10%); however, there were no significant differences between the studied groups regarding the distribution of symptoms (*P* > 0.05). Furthermore, there were significant differences between the studied groups regarding the site of the fissure (*P* = 0.005). Anterior and posterior were significantly higher among patients with partial sphincterotomy (*n* = 7, 35% and *n* = 10, 50%) than by patients with complete sphincterotomy (*n* = 2, 10% and *n* = 5, 25%). However, the lateral rate was significantly higher among patients with complete sphincterotomy (*n* = 13, 65%) compared with patients with partial sphincterotomy (*n* = 3, 15%) (Table 1).

**Table 1:** Demographic characteristics, medical history, distribution of symptoms, and distribution of site of fissure of the studied patients

Variables	Patients with partial sphincterotomy *N* = 20	Patients with complete sphincterotomy *N* = 20	Significance test	*P*-value
Gender				
Male	7 (35%)	9 (45%)	*χ*^2^ = 0.417	0.519
Female	13 (65%)	11 (55%)		
Age (years)	40.6 ± 7.80	42.85 ± 5.78	*t* = 1.035	0.307
No	16 (80%)	11 (55%)	*χ*^2^ = 6.459	0.264
Diabetes mellitus	2 (10%)	3 (15%)		
Hypertension	1 (5%)	2 (10%)		
Chronic constipation	1 (5%)	0 (0%)		
Laxative abuse	0 (0%)	3 (15%)		
Chronic constipation	0 (0%)	1 (5%)		
Pain	8 (40%)	8 (40%)	*χ*^2^ = 4.80	0.570
Bloody stool	1 (5%)	4 (20%)		
Difficulty passing stool	2 (10%)	2 (10%)		
Bleeding with defecation	4 (20%)	2 (10%)		
Chronic constipation	1 (5%)	2 (10%)		
Constipation	2 (10%)	2 (10%)		
Anal stenosis	2 (10%)	0 (0%)		
Anterior	7 (35%)	2 (10%)	*χ*^2^ = 10.694^a^	0.005*
Posterior	10 (50%)	5 (25%)		
Lateral	3 (15%)	13 (65%)		

Data are presented as mean ± SD, and frequency (%), and were analyzed using the chi-squared test (*χ*^2^) and independent *t*-test (t); *Significant

Additionally, there were significant differences between the studied groups regarding postoperative pain (*P* < 0.001). Postoperative pain was significantly higher among patients undergoing complete sphincterotomy (*n* = 14, 70%) compared with those undergoing partial sphincterotomy (*n* = 4, 20%). Furthermore, 19 patients with partial sphincterotomy achieved continence to flatus (95%), compared with 15 patients with complete sphincterotomy (75%). There were no significant differences between the groups studied regarding continence to flatus (*P* > 0.05). Additionally, there were significant differences between the studied groups regarding continence with hard stool (*P* = 0.037). Most patients with partial sphincterotomy continued to experience hard stools (*n* = 19, 95%), compared with 14 patients with complete sphincterotomy (70%). Moreover, there were significant differences between the studied groups regarding the occurrence of bleeding (*P* = 0.05). The occurrence of bleeding was significantly lower among patients who underwent partial sphincterotomy (*n* = 5, 25%) compared with those who underwent complete sphincterotomy (*n* = 11, 55%) (Table 2).

**Table 2:** Postoperative pain, continence to flatus, continence to the hard stool, and bleeding occurrence of the studied patients

	Patients with partial sphincterotomy *N* = 20	Patients with complete sphincterotomy *N* = 20	Significance test	*P*-value
Postoperative pain				
No	16 (80%)	6 (30%)	FE = 10.101	0.001*
Yes	4 (20%)	14 (70%)		
Continence to flatus				
No	1 (5%)	5 (25%)	FE = 3.137	0.77
Yes	19 (95%)	15 (75%)		
Continence to hard stool				
No	1 (5%)	6 (30%)	FE = 4.329	0.037*
Yes	19 (95%)	14 (70%)		
Bleeding				
No	15 (75)	9 (45)	*χ*^2^ = 3.75	0.050*
Yes	5 (25)	11 (55)		

Data are presented as frequency (%) and were analyzed using Fisher’s exact test (FE) and chi-squared test (*χ*^2^); *Significant

## Discussion

Chronic anal fissure (CAF) is a common and debilitating anorectal condition characterized by a painful tear in the anal canal, often associated with hypertonicity of the internal anal sphincter [9]. This increased sphincter tone leads to reduced blood supply to the anoderm, impairing wound healing and perpetuating the fissure [10, 11]. Partial LIS, where only a portion of the internal sphincter is incised, is proposed to balance fissure healing with a lower risk of postoperative complications, particularly fecal incontinence [12]. In contrast, complete LIS, involving full-length division up to the dentate line, is associated with higher healing rates but carries a greater risk of postoperative incontinence [9].

Fecal incontinence is a significant concern following LIS, as disruption of the sphincter mechanism may impair continence to gas and stool. Studies report variable rates of incontinence post-LIS, ranging from transient incontinence to flatus to more severe forms of stool leakage [13, 14]. A study by Nyam and Pemberton [5] found that 45% of patients who underwent total LIS experienced some degree of postoperative incontinence, while Liratzopoulos et al. [15] reported minor incontinence in 7.02% of cases at 48 weeks. However, other studies suggest that the risk of incontinence can be minimized with a tailored approach that limits the length of sphincter division to the extent of the fissure [11]. Given these controversies, this study aimed to compare the outcomes of tailoring the length of the lateral internal anal sphincter with either the whole length or the fissure apex and their effect on fecal continence in the treatment of chronic anal fissure.

The present study included 40 patients, with a predominance of female individuals (*n* = 24). Among them, 13 female patients underwent partial sphincterotomy, while 11 underwent complete sphincterotomy. The age of the studied patients ranged from 18 to 50 years, with a mean age of 40.6 ± 7.80 years in the partial sphincterotomy group and 42.85 ± 5.78 years in the complete sphincterotomy group. These findings align with the study conducted by Afifi et al. [16], who reported that CAF was most prevalent in the 30–50-year age group, with a mean age of 40.31 years, which is consistent with our results. Similarly, Davies et al. [17] found an average patient age of 49 years, supporting the trend observed in our study.

The female predominance observed in our study is also consistent with findings from ElGohary [9], who reported that 57.5% of patients with CAF were female, which is in agreement with our results. Likewise, Salih [18] determined a female-to-male ratio of 3.42 among patients who underwent surgery for CAF, and Afifi et al. [16] reported a similar ratio of 2.43. These findings reinforce the predominance of female patients observed in our study. Conversely, the study by Sarhan [19] reported only 22% female patients, which contrasts with our findings and suggests a possible geographical or demographic variation in the presentation of CAF. Additionally, Sarhan [19] noted that the peak incidence of CAF occurred in the fourth decade of life (55%), which is slightly older than the age distribution observed in our study and in studies by ElGohary [9] and Afifi et al. [16]. However, our results still fall within the broader range reported in the literature.

The current study demonstrated significant differences in the distribution of fissure sites between the studied groups. Anterior and posterior fissures were more prevalent among patients who underwent partial sphincterotomy (35% and 50%, respectively) compared with those who underwent complete sphincterotomy (10% and 25%, respectively). In contrast, lateral fissures were significantly more common among patients with complete sphincterotomy (65%) than in patients with partial sphincterotomy (15%).

These findings partially align with the study conducted by Marby et al. [20], who reported that the posterior midline was the most common site for anal fissures. Similarly, ElGohary [9] found that 78.75% of patients had their fissures located posteriorly, while 21.25% had them either anteriorly or both anteriorly and posteriorly. These results support the observation that posterior fissures are generally more common, which is consistent with our findings in the partial sphincterotomy group. However, the significantly higher proportion of lateral fissures among patients undergoing complete sphincterotomy in our study differs from these previous findings. This variation may be attributed to differences in patient selection, underlying pathophysiological mechanisms, or variations in surgical techniques. The higher prevalence of lateral fissures in patients undergoing complete sphincterotomy might suggest that certain anatomical or functional factors influence fissure location in patients undergoing different types of sphincterotomy. Further studies are needed to explore these variations and their clinical implications.

The present study revealed significant differences between the studied groups in terms of postoperative pain and bleeding occurrence. Postoperative pain was significantly higher among patients who underwent complete sphincterotomy (70%) compared with those who underwent partial sphincterotomy (20%). Similarly, bleeding was significantly more common in the complete sphincterotomy group (55%) than in the partial sphincterotomy group (25%).

Our findings contrast with those of ElGohary [9], who reported that postoperative pain and bleeding were significantly reduced with full-length LIS compared with partial-length LIS. Additionally, Pujahari [21] found that bilateral LIS significantly reduced pain, reporting a pain score of 12 out of 100 compared with 57 in unilateral LIS, even with an average dose of nonsteroidal anti-inflammatory drugs (NSAIDs). These studies suggest that the extent of sphincterotomy can influence pain outcomes, though differences in technique or patient populations may explain the discrepancy with our results. Conversely, in studies by Acar et al. [10] and Sabuncuoglu et al. [22] evaluating postoperative pain relief at 1, 4, and 6 weeks, VAS-reported pain relief rates were between 91.4% and 100%. Sabuncuoglu et al. [22] noted that although pain relief was significant, open LIS had a slightly higher pain rate (8.7%) than closed LIS (5.1%), though this difference was not statistically significant. These findings suggest that while pain relief is generally achieved following LIS, variations in surgical technique and follow-up duration may contribute to differences in reported pain levels.

In terms of bleeding, our findings are inconsistent with Javed [11], who reported that none of the patients in either group experienced postoperative bleeding after 1 week of follow-up. Similarly, Moosavi et al. [23] observed that 100% of patients treated with either procedure did not exhibit postoperative pain or bleeding. These results suggest that bleeding incidence may depend on surgical technique, postoperative care, or individual patient factors. Postoperative complications, including bleeding, infection, and incontinence, are well-documented concerns after LIS. Kang et al. [24] identified these as potential complications, with Pernikoff et al. (1994) reporting a 15% postoperative bleeding rate, which is lower than what we observed in our complete sphincterotomy group. However, Afifi [16] reported a significantly lower bleeding rate of 1.8%, suggesting variations in patient management. Infection rates were also low, with Davies et al. [17] reporting a 1.3% infection rate and Afifi [16] reporting 2.5%, which may be attributed to differences in postoperative hygiene practices.

The current study found that continence to flatus was preserved in 95% of patients who underwent partial sphincterotomy compared with 75% of those who underwent complete sphincterotomy, although this difference was not statistically significant. However, significant differences were observed in continence to hard stool, with 95% of patients undergoing partial sphincterotomy maintaining continence, compared with 70% in the complete sphincterotomy group.

Our findings align with ElGohary [9], who reported that full-length division of the internal sphincter was associated with a higher incidence of temporary incontinence to flatus and stool, despite offering better long-term results than partial-length LIS. Additionally, Kensarah et al. [12] observed incontinence rates of 14.1% for flatus, 4.3% for liquid stools, and 1% for solid stools in patients who underwent partial LIS, further supporting the notion that sphincter division extent influences incontinence risk. Other studies have reported variable rates of incontinence after LIS. Arroyo et al. [13] documented a 92.5% recovery rate after LIS, with a 5% rate of procedure-related anal incontinence, a finding echoed by Sánchez et al. [25]. Similarly, Acar et al. [10] reported gas/liquid incontinence in 1.8% of patients, with all cases occurring in women with a history of vaginal delivery. These studies highlight the potential risk factors contributing to incontinence beyond surgical technique alone.

Our study showed lower rates of incontinence compared with some historical reports. For instance, Mate [26] found that incontinence to flatus occurred in 8% of LIS patients, with no fecal incontinence reported, while Watts et al. [27] documented flatus incontinence in 19% and fecal incontinence in 8% of LIS patients. Shaikh et al. [14] observed flatus incontinence in 1.28% of LIS patients, a rate significantly lower than that found in older studies. Lewis et al. [28] reported an overall incontinence rate of 17%, while Nelson et al. [29] estimated the risk of incontinence post-sphincterotomy at 10%. These findings highlight that while LIS is effective, the extent of sphincter division and patient-specific factors can significantly influence incontinence outcomes. Despite these variations, several studies suggest that judicious surgical techniques and familiarity with anorectal anatomy can minimize complications [30]. Malik et al. [30] reported transient flatus incontinence in 64.3% of patients, resolving within 2 months, and permanent fecal incontinence in 7.1%. Other studies, such as Garcea et al. [31], found flatus incontinence in 1.7% of partial LIS patients.

The study’s limitations included a short follow-up duration, the inclusion of only patients without prior anal conditions, and a relatively small sample size.

## Conclusions

This comparative study highlights that while both partial and complete LIS are effective in resolving CAF, complete LIS is associated with a higher risk of postoperative fecal incontinence, particularly for flatus and liquid stools. Partial LIS, however, appears to offer a balance between fissure healing and continence preservation, making it a preferable option for patients at higher risk of incontinence. The findings suggest that tailoring the extent of sphincter division on the basis of individual patient factors, such as baseline sphincter function, age, and prior anorectal surgeries, may optimize outcomes.

## Data Availability

The data are available upon reasonable request from the corresponding author.
